# State and evolution of the African rainforests between 1990 and 2010

**DOI:** 10.1098/rstb.2012.0300

**Published:** 2013-09-05

**Authors:** Philippe Mayaux, Jean-François Pekel, Baudouin Desclée, François Donnay, Andrea Lupi, Frédéric Achard, Marco Clerici, Catherine Bodart, Andreas Brink, Robert Nasi, Alan Belward

**Affiliations:** 1 Institute for Environment and Sustainability, Joint Research Centre of the European Commission, Ispra, Italy; 2 Earth and Life Institute, Université catholique de Louvain, Louvain-la-Neuve, Belgium; 3 Food and Agriculture Organisation, Rome, Italy; 4 Centre International for Forest Research, Bogor, Indonesia

**Keywords:** deforestation, earth observation, African rainforests

## Abstract

This paper presents a map of Africa's rainforests for 2005. Derived from moderate resolution imaging spectroradiometer data at a spatial resolution of 250 m and with an overall accuracy of 84%, this map provides new levels of spatial and thematic detail. The map is accompanied by measurements of deforestation between 1990, 2000 and 2010 for West Africa, Central Africa and Madagascar derived from a systematic sample of Landsat images—imagery from equivalent platforms is used to fill gaps in the Landsat record. Net deforestation is estimated at 0.28% yr^−1^ for the period 1990–2000 and 0.14% yr^−1^ for the period 2000–2010. West Africa and Madagascar exhibit a much higher deforestation rate than the Congo Basin, for example, three times higher for West Africa and nine times higher for Madagascar. Analysis of variance over the Congo Basin is then used to show that expanding agriculture and increasing fuelwood demands are key drivers of deforestation in the region, whereas well-controlled timber exploitation programmes have little or no direct influence on forest-cover reduction at present. Rural and urban population concentrations and fluxes are also identified as strong underlying causes of deforestation in this study.

## 1. Introduction

Rainforests cover only 13% of Africa's landmass [1], but they account for more than 90% of the carbon stored in the continent's terrestrial ecosystems [2,3], provide the habitat for many plant and animal species ([4]; table 1) and play a significant role in the climate system [5]. Africa's forests support the direct livelihood of 60 million rural people (providing food, medicine, fuel, fibre, non-timber forest products as well as social and cultural functions) and less directly support 40 million people living in urban centres in the forest domains [6]. Africa's forests, which make up around 20% of the total global tropical rainforest area [7], are mainly concentrated in the Congo Basin; after the Amazon basin, this is the world's second largest contiguous forest zone. The ecological zonation proposed by the United Nations Educational, Scientific and Cultural Organization [8] has three African ecoregions dominated by rainforests: Guineo-Congolian (in West and Central Africa), East Malagasy (Madagascar) and AfroMontane (Central and Eastern Africa).

**Table 1.** Total number of species (mammals, birds, amphibians) and total carbon stock [2] in each of the three regions. The columns ‘threatened’ report the percentage of species falling in the following categories of the IUCN Red list: Critically Endangered, Endangered, Vulnerable [4].

	mammals		birds		amphibians		carbon	
	total	threatened (%)	total	threatened (%)	total	threatened (%)	total (GTonnes)	% Africa
Central Africa	493	11.4	1100	1.4	288	15.3	39.2	78.5
West Africa	355	13.2	825	1	193	18.1	5.8	11.6
Madagascar	159	20.8	217	6	219	11.9	1.8	3.6

Mapping the extent and monitoring the state of African rainforests is of paramount importance because their location and condition affect the wellbeing of millions of rural and urban people, affect the regional and global climate, and have significant consequences for biodiversity. Accurate delineation of forest area and composition along with documentation of dynamics (such as change in area, inter- and intra-annual seasonal patterns, carbon content and other geobiophysical variables) provides information for science (e.g. reducing uncertainty in the carbon cycle) and for policy (e.g. formulation, implementation, monitoring, reporting and verification). Forest area and condition information establishes the boundary conditions for hydrological and geochemical cycle studies and for climate models, yet policy users need the same information to formulate and evaluate sustainable development policies. This nexus is seen at local and global scales in multilateral environmental agreements such as the United Nations Framework Convention on Climate Change (UNFCCC), where policies such as reducing emissions from deforestation and degradation (REDD+) recognize the role of forests in the carbon cycle; and the convention on biological diversity (CBD), which identifies forest habitat loss as a critical driver of declining biological diversity. While global in scope, the processes of reporting and verification of policy linked to UNFCCC and CBD often require local information on land cover and land-cover changes; in other words, we need to monitor the forests on local scales globally.

Land-cover information is also needed to measure the effectiveness of management associated with sustainable development. Addressing issues such as logging, forest conservation and restoration, agricultural land expansion, desertification or watershed degradation will all substantially benefit from the availability of accurate baseline forest-cover information. Indeed, the Observatory for Central African Forests (OFAC), under the auspices of forest ministries in the region, now report on the ‘state of the forest of the Congo Basin’ every 2 years [9,10].

Baseline forest-cover information was originally derived from a combination of aerial photography and field surveys. The thematic descriptions attached to the resulting classifications were detailed, but the spatial output had limited accuracy, especially when aggregated at the continental level [8]. The advent of earth-observing satellites led to improvements in the spatial integrity and detail of the maps while retaining levels of thematic content [11]. Since the turn of the twenty-first century, it has been possible to generate daily images (albeit complete with clouds) of the entire continent from satellite systems such as Vegetation (VGT, with 1 km resolution), the Medium Resolution Imaging Spectrometer (MERIS, at 300 m) and Moderate Resolution Imaging Spectroradiometer (MODIS, at 1 km to 250 m). Global land cover mapping exercises, such as the MERIS-based GlobCover 2005, include maps of Africa's rainforests. But GlobCover's Central African mapping is compromised by a limited number of cloud-free image acquisitions over the region. More localized products including continental maps [1] and regional products [12] are based on a greater number of satellite overpasses, and therefore more opportunities for cloud-free observation, but these data are still acquired at a coarse resolution of 1 km. Access to higher-resolution satellite imagery has improved since free and open access was provided to the Landsat archives. Using these data, continental- and global-scale sample-based surveys measuring forest cover [13] and forest-cover change at resolutions of 30 m are now increasingly undertaken. The European Commission's Tropical Ecosystem Environment observation by Satellite (TREES) project is one example. This project, which has been funded since 1992, uses Thematic Mapper (TM) and enhanced TM data from Landsat satellites [14] to provide sample-based information on changes in the state of the world's tropical forests [15,16]. The TREES project currently addresses change over three epochs, 1990, 2000 and 2010, and contributes to the remote sensing survey of the Food and Agriculture Organisation's Forest Resource Assessment (FAO-FRA) for 2010 [17].

This paper presents a new wall-to-wall forest map covering all three of Africa's rainforest blocks at 250 m resolution using daily observations from one single year, 2005. The new maps are accompanied by measures of deforestation made at 30 m resolution between 1990, 2000 and 2010. Regional drivers of forest-cover change are also identified and attributed on the basis of available geospatial data and statistics.

## 2. Material and methods

### (a) Wall-to-wall forest mapping

Using data from MODIS sensors on both the Terra and Aqua satellites, we assembled four MODIS daily products from collection version 5 for surface reflectance at 250 and 500 m spatial resolution. Three spectral bands were used: the red and the near-infrared (NIR) at 250 m spatial resolution and the middle infrared (MIR) at 500 m spatial resolution. The imagery covered the entire African continent for the year 2005.

Production of cloud-free datasets is a significant challenge to mapping land-cover types in this equatorial region. Quality flags indicating cloud cover and missing data accompany the daily MODIS surface reflectance images. Two satellites acquiring data increase the chance of cloud-free imaging and so we used daily data from both the Aqua and Terra missions. We then excluded cloudy/bad data using the mean compositing strategy [18,19]. This averages cloud-free reflectance values [20] to create an annual cloud-free composite image for 2005. This compositing method reduces directional reflectance effects and limits any remaining atmospheric perturbations and cloud contamination. The MIR channel was resampled using the nearest-neighbour rule to match the 250 m of the red and NIR channels to generate a final three-band mosaic output.

As a first classification step, we used an empirically derived Normalized Difference Vegetation Index (NDVI) threshold to identify and exclude open dry forests, savannas, bare soils and water surfaces. Then, an unsupervised classifier, ISODATA, was run on the red, NIR and MIR bands of the remaining data. The resulting classes were interpreted based on field knowledge, reference maps [1,21,22], high spatial resolution images (better than 5 m) and temporal profiles from our 730 daily MODIS images from 2005. These results were then clustered into four final classes: lowland rainforest (more than 70% of tree cover on non-flooded soil), swamp forest (flooded forests with more than 70% of tree cover), rural complex (10–30% tree cover and more than 50% croplands) and other land cover (any remaining savanna, croplands … ). Classification accuracy was then assessed by an independent expert, using high-resolution imagery (1–4 m) from Google Earth as a surrogate for ground data [23]. In total, 320 points were interpreted in the four land-cover classes.

### (b) Forest-cover change assessment

To quantify changes in forest cover, we followed the TREES/FAO-FRA approach [24]. This uses Landsat image extracts of 10 × 10 km from a systematic sample on each latitude/longitude degree intersect over three epochs: 1990, 2000 and 2010. This gave a total sample of 285 points systematically distributed across Africa's rainforests. Of these, 256 sites were covered by good-quality image pairs between the various epochs: 173 over Central Africa, 67 over West Africa and 16 over Madagascar. The 28 missing samples were mostly located in cloudy areas over the coastal regions of Gabon, Equatorial Guinea and Cameroon.

While our target epochs were 1990, 2000 and 2010, persistent cloud cover, limited data acquisitions and satellite failures required each epoch to be constructed from imagery spanning some years either side of the target year. The mean acquisition date and range for the three epochs are respectively 11 August 1987 (1984–1994), 31 March 2001 (1996–2003) and 28 March 2010 (2008–2011). Image acquisition was particularly limited for the 2010 epoch following technical problems with Landsat 7 post-2003. In consequence, the 2010 epoch was constructed using Landsat plus additional satellite sources; Disaster Monitoring Constellation (DMC) accounted for 43% of the images used, SPOT-4 and 5 HRV (15%) and Landsat TM the remaining 42%.

Forest-cover changes over the period 1990–2000–2010 were then estimated using the TREES object-based processing chain. This includes different steps using multi-date Landsat imagery [25,26]: visual screening of best available images from the entire Landsat open archive, co-registration, calibration, cloud and shadow masking, segmentation, change detection and classification [25,27]. This processing chain was adapted for the DMC and SPOT samples used in the 2010 epoch [28]. The minimum mapping unit was 5 ha. The final legend used the following aggregation rules: tree cover (at least 70% tree cover portion in segment), tree cover mosaic (30–70% tree cover portion), other wooded land (at least 70% shrubs, forest regrowth), other vegetation cover (including croplands, herbaceous and bare lands) and water. We defined deforestation as the conversion of tree cover and half of the tree cover mosaic into one of the other land-cover classes: reforestation and/or afforestation were defined as the opposite. Experts from each region then checked the resulting land-cover maps, and recoded classes wherever they encountered misclassifications from the automatic chain. Forest-cover change could then be measured by comparing the quality-controlled image pairs between 1990 and 2000 and between 2000 and 2010 at each degree sample location.

Four correction steps were applied to give the same sampling probability to each site and respect the reference dates. First, as images were acquired at different dates around our epoch target years, the land-cover changes matrices were linearly adjusted to a common reference date of 30 June for 1990, 2000 and 2010. This is based on the assumption that land-cover change rates are constant during any given period [16]. Second, any remaining cloudy areas in a particular sample were considered as an unbiased loss of data, and assumed to have the same proportions of land cover as the non-cloudy areas within the same site. This was achieved by converting the land-cover change matrices to area proportions relative to the total cloud-free land area of the sample site. Third, where sample sites were completely missing, we used a local average from surrounding sample sites as surrogate results. Fourth, we had to correct for changing sample probability with latitude; our sample sites are taken at each degree latitude/longitude intersection, and owing to the earth's curvature, the sampling probability increases with latitude, which leads to higher-intensity sampling in higher latitudes. To correct for such unequal sampling probability, sample sites were given a weight equal to the cosine of the latitude for producing the land-cover change matrix over the whole region.

For the study area, the total land-cover area can be extrapolated from the average proportion using the Horvitz–Thompson direct expansion [16,29,30]. The total class area *Z*_c_ is obtained from:
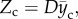
where *D* is the total area of the study region. The standard error (s.e.) is then calculated as:
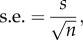
where *n* is the total number of available sample sites and *s* is an estimator of the s.e. based on local variance estimation.

Finally, the annual change rates were calculated by dividing the total change area by the time period and by the total cover area averaged between the two dates.

### (c) Deforestation drivers and underlying factors

To assess the relative importance of drivers of deforestation in Africa, we followed Geist & Lambin's theoretical frame [31]. A series of geospatial datasets documenting recognized drivers of deforestation (agricultural expansion, infrastructure development, timber extraction and fuelwood extraction) were used along with evidence concerning underlying causes (demographic pressure, economy, political instability and governance). We also considered the efficiency of designated protected areas as protection against deforestation. Statistical analysis of these with reference to the individual sample points used for the forest-cover change assessment described above could then be carried out. However, the paucity of reliable geospatial data associated with the known drivers imposed some limitations to this.

Timber extraction statistics were available for logging concessions reporting to OFAC [10], and official protected area boundaries were available from IUCN [32]. However, too few of our forest-cover sample points from the systematic sample grid intersected with either logging concession or protected areas to compute reliable statistics. In this case, we support our analysis using a localized but more intensely sampled study in Central Africa [33].

More complete datasets could be obtained for agricultural expansion, as gridded information on cropland proportions was available [34]. Infrastructure development (principally road networks and urban development) datasets were incomplete. However, the spatial pattern of cropland areas in Central Africa follows old road networks [11], and so the cropland analysis, in part, integrates road density. This is particularly evident in the Democratic Republic of Congo (DRC) where cropland mosaics match road networks established in the colonial period. Infrastructure elements were also incorporated into our metric concerning influence of urban populations, which we approximate through the travel time to the closest city of more than 50 000 people [35]. Finally, we extracted the rural population from population density maps [36].

To reduce the impact of pre-existing deforestation on subsequent analysis, we first eliminated forest-cover sample points with less than 20% forest cover. We then extracted the deforestation rate for each of the remaining 150 sample points and combined this with the following metrics: area covered by croplands, travel time to cities and population density. Then, we conducted statistical analysis separately for each parameter. We ranked the entire population of samples by increasing value of each parameter and we divided the entire dataset into six homogeneous groups of 25 samples. An analysis of variance was then conducted over the deforestation area observed in the six groups separately for each parameter.

## 3. Results

### (a) Forest mapping

**Figure 1.**
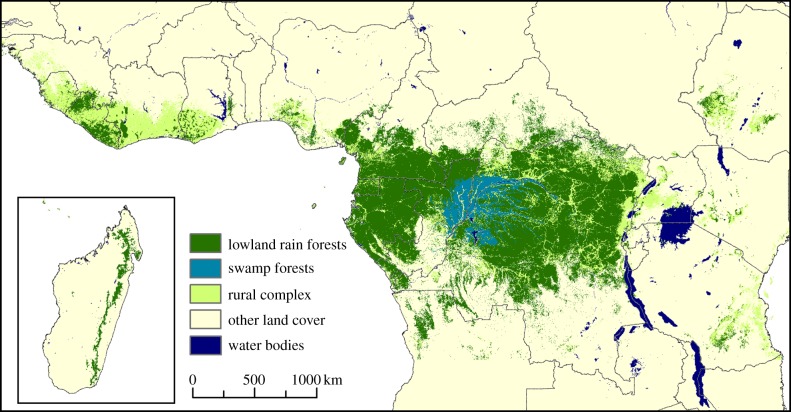
Spatial distribution of the African rainforests derived from MODIS data.

Figure 1 shows the spatial distribution of Africa's rainforests. Independent validation of the map indicates an overall accuracy of 83.75%, with users' and producers' accuracies of 87% and 85.2%, respectively, for the lowland rainforest, and 81.6% and 98% for swamp forest. Table 2 provides the forest-cover area by country. Central Africa accounts for 89% of Africa's rainforests, and the DRC alone contains some 53.6%. East African countries with Afromontane forests (Ethiopia, Kenya, Tanzania and Uganda) represent only 2% of the rainforests—though these countries do have large areas of dry forest [1].

**Table 2.** Forest area for the large forested African countries (and total area for the main regions in italic), estimated from the MODIS-derived map.

country	humid rainforests (×1000 ha)
Democratic Republic of Congo	107 181
Gabon	22 416
Congo	20 932
Cameroon	20 037
Central African Republic	5833
Equatorial Guinea	2163
*Central Africa*	*178 564*
Liberia	4552
Nigeria	3158
Cote d'Ivoire	1530
Ghana	1487
other countries	1273
*West Africa*	*12 002*
*Madagascar*	*4385*
*Eastern Africa*	*4876*
*total Africa*	*199 829*

**Figure 2.**
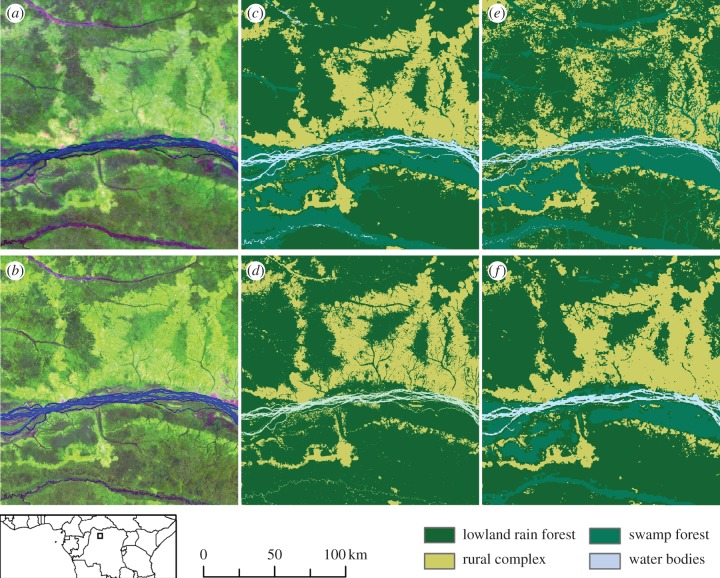
Comparison of the satellite images and forest-cover maps over the region of Lisala-Bumba (DR Congo). (*a*) MODIS colour composite; (*b*) Landsat colour composite; (*c*) MODIS-derived map (our study); (*d*) Landsat-derived map [13]; (*e*) GlobCover map [22] and (*f*) synthesis map [12].

Results of our map's thematic and spatial attributes are also examined through visual comparison with previously published maps [12,13,37], shown in figure 2. As expected, the Landsat-derived map [13] shows finer spatial resolution and depicts detailed linear features, but does not distinguish swamp from lowland forests. Comparison with the MERIS-based GlobCover 2005 highlights the benefit of our data stream from two satellites' overpasses per day in reducing cloud contamination; the cloud-induced salt-and-pepper effect evident in GlobCover 2005 is absent from our new map. The synthesis map [12] is very similar to our product in this region, whereas in coastal Cameroon and Congo the main source of data used for the mapping had a 1 km resolution, which clearly impairs the spatial quality of the map.

### (b) Forest monitoring

**Figure 3.**
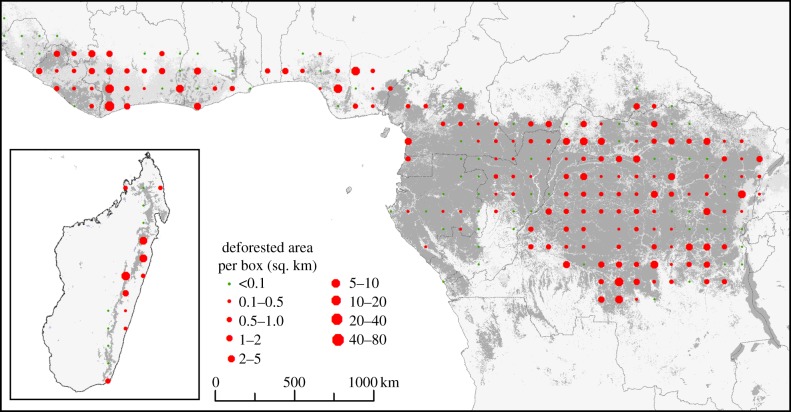
Net deforestation between 1990 and 2000. The circle size is proportional to the surface affected by deforestation in each sample of 100 km^2^.

**Figure 4.**
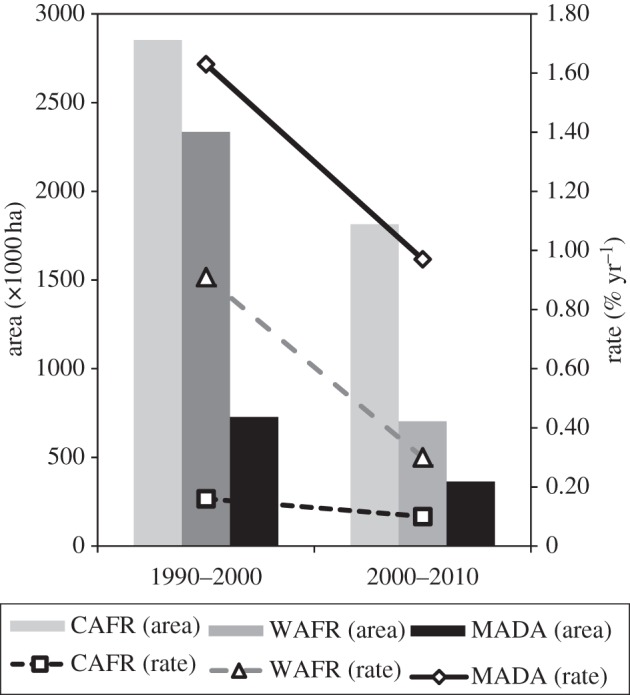
Evolution of the deforestation area and annual rate between the two epochs for the three regions (CAFR, Central Africa; WAFR, West Africa; MADA, Madagascar).

Table 3 shows estimates of net deforestation for Africa's three main rainforest regions. Figure 3 illustrates deforestation ‘hot spots’ for West Africa, Madagascar and the fringes of the Congo Basin. Africa lost 0.59 million hectares of rainforest annually between 1990 and 2000, which decreased to 0.29 million hectares a year between 2000 and 2010. Central Africa accounts for 50–60% of the total deforested area, but the annual deforestation rates *per se* are much lower than in the other two regions (table 3). The initial forest area used for the estimates of the rates is extrapolated from the samples, and can differ slightly from the area measured on the forest-cover map owing to cloud-cover and missing samples. In all three regions, deforestation decreased by between 37% and 67% between 2000 and 2010 with respect to the 1990–2000 period (figure 4). Madagascar is an exception, and deforestation in this region remains high. However, note that the limited number of samples in Madagascar increases the confidence interval for the results. To a lesser extent, this also holds true in West Africa. It is also evident that our systematic sampling method is not optimum for measuring deforestation in the highly fragmented pattern of forest patches so characteristic of the Afromontane domain.

**Table 3.** Gross and net deforestation areas over 1990–2000 and 2000–2010 and annual rates measured from the sample of satellite images, for the three regions and the two epochs (areas in 1000 ha). The numbers in parentheses represent the standard deviation of the estimates.

	1990–2000				2000–2010			
	gross deforestation	annual rate (%)	net deforestation	annual rate (%)	gross deforestation	annual rate (%)	net deforestation	annual rate (%)
Central Africa (*n* = 173)	345.9 ± 54	0.19	285.4 ± 36.5	0.16	187.6 ± 22.2	0.11	181.5 ± 39.8	0.10
West Africa (*n* = 67)	278.7 ± 77.9	1.09	233.5 ± 108.3	0.91	82.1 ± 14.1	0.35	70.4 ± 23.9	0.30
Madagascar (*n* = 16)	75.8 ± 25.8	1.69	728 ± 32.8	1.63	40.5 ± 18.2	1.08	36.4 ± 24.8	0.97
total three regions	700.4	0.33	591.9	0.28	310.2	0.15	288.3	0.14

At 0.59 million ha yr^−1^, the area of deforestation for Africa for the period 1990–2000 may seem large. However, this is four times smaller than the area lost in Latin America [38]; at 0.3% yr^−1^, the rate of loss too is lower for Africa than Latin America, with the latter losing its forest at around 0.4% yr^−1^.

### (c) Deforestation drivers and underlying factors

Expansion of cropland area, increasing urban population and associated expansion of urban infrastructure all bring forested areas into closer proximity with urban boundaries. This, in turn, increases human access to forested areas. With high levels of significance (*p* < 0.001), our analysis confirms all three processes as key drivers of deforestation.

**Figure 5.**
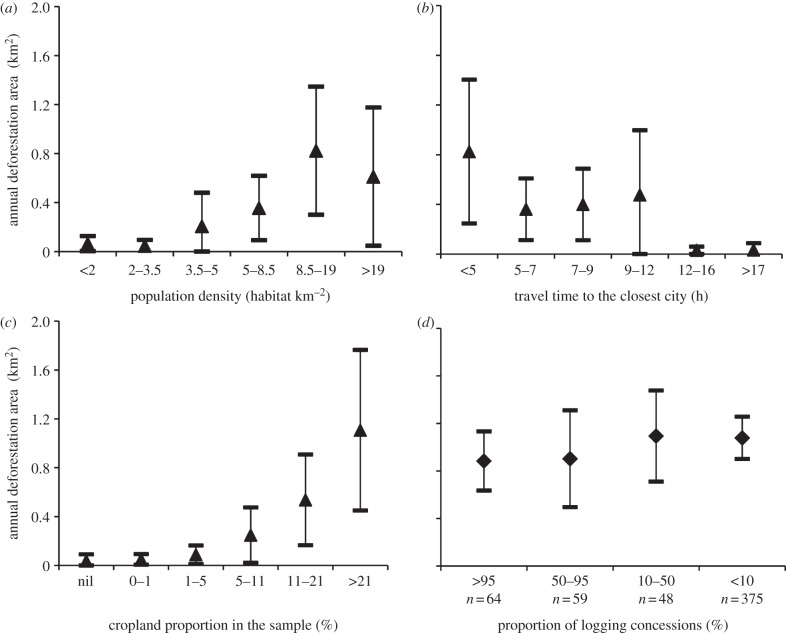
Relationship between the deforestation observed by homogeneous group of samples according to the following parameters. (*a*) Population density [36]; (*b*) travel time to closest city [35]; (*c*) area covered by croplands [34] and (*d*) area covered by logging concession [33].

Figure 5*a* shows that deforestation dramatically increases when rural population density exceeds 8.5 people per km^2^.

The influence of urban population on deforestation is approximated by travel time to the closest city of 50 000 people [35]. Figure 5*b* shows a regular decrease in deforestation with increasing travel times up to 6 h. This slows slightly from 7 to 16 h of travel time, and deforestation is close to zero for samples at more than 13 h of travel time.

Figure 5*c* illustrates the strong relationship between cropland proportion and deforestation. Deforestation increases as soon as cropland proportion reaches 5% of the sample, and this relationship becomes extremely important once cropland proportion exceeds 20%.

Figure 5*d* shows the relationship between deforestation rates and the area of logging concessions. The intensity of logging concessions does not appear to accelerate deforestation rates in this region.

## 4. Discussion

### (a) Forest mapping

Table 2 reaffirms the dominance of the Congo Basin concerning Africa's rainforest distribution. This largely coherent forest area in Central Africa contrasts markedly with the more fragmented forests in West Africa, East Africa and Madagascar. The patterns of this fragmentation differ from region to region. In East Africa, forest-cover fragmentation is linked to topography; the dense humid forests follow the high ground in the mountainous areas. In West Africa, the forest patches are the remnants of a more massive block, which has been heavily deforested since the middle of the twentieth century [39]. In Madagascar, the linear pattern results from the conjunction of topography and intense deforestation owing to agriculture and mining.

### (b) Forest monitoring

On the basis of our estimates, the Congo Basin shows a lower deforestation rate than other tropical forest regions of the world [15]. In consequence, the contribution of Africa to carbon dioxide emissions owing to deforestation is limited with respect to southeast Asia and Latin America, and the future REDD+ mechanisms must take into account this particularity [40].

With rare exceptions in West Africa, we do not observe massive deforestation over large contiguous areas planned for huge agro-business plantations (oil palm, rubber).

The deforestation hot spots observed between 1990 and 2010 in this study, based on a systematic sample of satellite images, correspond extremely closely to an earlier map of hot spots based on experts' opinion [41]. In all three African regions, deforestation has slowed down post-2000, though continues to remain higher in West Africa and Madagascar than in Central Africa. These regional differences could, in part, be explained by differential population pressure: the population density in Central Africa for samples with more than 20% forest cover is two times lower than in Madagascar and eight times lower than in West Africa.

A comparison was conducted with results of Potapov *et al*. [13] on DRC, where a deforestation of 0.25% yr^−1^ was measured between 2000 and 2010 for humid forests, which is slightly higher than our estimates on the entire basin, but other studies showed a higher deforestation rate in DRC than in the other Congo Basin countries [42].

### (c) Deforestation drivers and underlying factors

Geist & Lambin [31] considered three main causes of deforestation to be agricultural expansion, wood extraction and infrastructure. Their results showed that expansion of agricultural lands was the lead direct cause of deforestation but also that no single cause ever operated alone, stating ‘it is a striking feature of reported cases that no causation by single variables, but rather synergistic factor combinations are important’ [31, p. 23]. Their sample for the Congo Basin region was, however, very limited (two cases in southeast Cameroon, one case in northeast and one in southeast DRC). With our much larger systematic sample, we can empirically identify the main drivers of deforestation in our region. Our data allow us to highlight the role, importance and thresholds of two major combinations: (i) rural population, shifting agriculture and logging, and (ii) urbanization and fuelwood extraction.

#### (i) Rural population, shifting agriculture and logging

As shown in figure 5*c*, deforestation becomes significant once croplands account for around 10% of the area in 2000 and increases sharply above this threshold. Cultivation follows two main patterns in the Congo Basin: ‘diffuse’ (characterized by small, dispersed openings corresponding to traditional shifting cultivation) and ‘corridor’ (small-to-medium contiguous openings, which follow the road network linking villages and human settlements—a result of the policies from colonial powers continued by independent states). We have anecdotal evidence that the diffuse pattern is getting less and less common, whereas corridor patterns now dominate. Previous work [31] has established that agriculture does not act alone and that the ‘agriculture–population’ combination is a prominent cause of deforestation. With a population growth rate of between 1.8% yr^−1^ in Central African Republic and 2.8% yr^−1^ in DRC, the demand for agricultural land will drastically increase in the next 20 years (unless technology for agricultural intensification significantly improves) and, unfortunately, agricultural land is likely to expand at the expense of forest [43]. Our data show that deforestation drastically increases when rural population exceeds 8.5 people per km^2^ (figure 5*a*). This result is consistent with earlier work in Central Africa that identified a population density threshold of eight people per km^2^ as a value above which deforestation rates accelerated [33].

A population density of 10–15 persons km^−^² is reached in many forested areas through the intrinsic increase in population and because of the migration of rural populations towards cities. People move closer to roads to benefit from improved access to markets (as well as other elements of urban infrastructure such as schools and healthcare). This population density increase is incompatible with traditional shifting cultivation practices and long fallow periods. Reducing shifting cultivation theoretically reduces new clearings of primary forests, but it also curtails regrowth on fallow land, because the land is never left fallow. This, in turn, reduces soil fertility, which leads to food insecurity [44]. But as population density continues to increase and agricultural intensification fails to take hold, so actual deforestation and degradation will continue as the need for more cropland rises [45]. In the near future, the increasing international demand for commodities such as coffee, cocoa, rubber or oil palm is also likely to increase the demand for agricultural land. Given the evident dominance of the corridor pattern of deforestation, we can reasonably conclude that road network density is an important factor. As the presently limited road network expands (for whatever purpose, be it logging, mining or linking settlements), this will render large blocks of currently inaccessible forests accessible—and access to forests has been identified as a key factor facilitating deforestation [46]. In the Congo Basin, the most rapidly changing area has been northern Congo, where the rate of road construction increased from 156 km yr^−1^ for the period 1976–1990 to over 660 km yr^−1^ after 2000 [47], as shown in figure 3. It is possible to control access to logging or mining roads and to mitigate part of the threat but not to remove it completely—a complete ban on road building would significantly reduce the development prospect of many remote areas.

We have shown that samples with a population reaching a threshold of 8.5 people km^−2^, and/or an area of agricultural land above 10% associated with the road network were significantly more deforested than samples below these thresholds. We need to add wood extraction to these factors. In rural areas, wood is extracted for construction and service (poles) and for woodfuel (fuelwood usually, sometimes charcoal)—neither are considered important deforestation factors in the literature, yet logging is. This assertion is not supported by our results. Figure 5*d* shows that for our samples deforestation does not vary as a function of the area of occupied by logging concessions. This relatively low impact of logging operations on deforestation can be due to the recent efforts of the Congo Basin countries towards sustainable forest management [48], a view also confirmed by other studies [49]. The first of Central Africa's forest management plans was implemented by Cameroon in 2000, which currently has over 14 million ha of forest concessions managed in accordance with state-approved plans [6]. Certification has also demonstrated significant progress in Gabon, Cameroon and Congo, from zero certified forests in 1995 to 4.8 million hectares in 2010 [10]. International initiatives promoting legal exploitation also have a positive effect on forest management. However, the opening of roads in a completely intact forest area may provoke deforestation sometime in the future; monitoring such ‘delayed deforestation’ requires datasets spanning many years.

#### (ii) Urbanization, permanent agriculture and fuelwood extraction (charcoal)

We approximated the influence of urban populations using travel time to the closest city of 50 000 people or more [35]. Figure 5*b* shows a clear pattern with two apparent inflexion points: deforestation between 2000 and 2010 was very high when less than 5 h from a city, almost nil when more than 12 h from a city and stable in between. The curve illustrates the ‘island’ pattern of deforestation described by Mertens & Lambin [50] for peri-urban areas. Five hours corresponds to a daily return trip and could be considered as a permanently deforested (either cultivated or extensively degraded) circle around the city. The plateau between 5 and 12 h could be interpreted as a combination of shifting agriculture and a provisioning area that supplies woodfuel to the city. In sub-Saharan Africa, wood remains the main source of domestic energy and meets over 80% of needs across all countries [51]; in DRC, wood energy constitutes more than 95% of total wood production, with an annual consumption estimated to be 1 m^3^ per person [52] and a total production higher than 70 million m^3^. Forests, most notably peri-urban forests, play a key role in providing woodfuel, with charcoal being the fuel of choice, given that it is much easier to stock, transport and manipulate than fuelwood. The consumption of charcoal for the city of Kinshasa is estimated to be the equivalent of 4.8 million m^3^ of wood, which affects huge forest areas up to a distance of 300 km from the city [52].

We have shown the importance of travel time to urban areas in determining deforestation rates, with thresholds at 5 and 12 h. Future development plans in the region are likely to include improvements to road and transport infrastructure. While laudable from many economic perspectives, such improvements will reduce travel times to cities from surrounding regions. Given the pattern shown by our data, this implies a much bigger threat to remaining forests than envisioned from the prevailing corridor patterns alone.

## 5. Conclusion

The mapping and monitoring of Africa's rainforests is an ongoing process, where new techniques and new data continuously improve our knowledge. Unfortunately, threats to these precious and unique resources also appear equally ongoing. In this paper, we exploit newly available images and advanced image processing techniques to derive accurate forest cover and forest-cover change estimates. However, sensors with better radiometric quality (Landsat 8) and finer spatial resolution (Sentinel-2) will soon be available, which will allow us to further refine and improve both maps and measurements. Acquiring data from as many satellite overpasses as possible directly at African receiving stations should also improve access to cloud-free images. Many initiatives collect information on the ground, which can help improve our understanding of deforestation drivers. However, further effort is needed to link spatial modelling with multivariate analysis analysing deforestation and the different drivers.

African rainforests provide vital resources for local populations. They support the national economies of many countries and deliver key global ecosystem services, including climate regulation and biodiversity. Future research should aim at improving our understanding of the interactions between the local, national and global scales and a range of thematic issues. In the long term, the international community has to build strong African capacities in order to give the Continent all the winning cards for defining and implementing sound forest management policies.
